# Characterization of bacteria swarming effect under plasmonic optical fiber illumination

**DOI:** 10.1117/1.JBO.28.7.075003

**Published:** 2023-07-18

**Authors:** Jang Ah Kim, Yingwei Hou, Meysam Keshavarz, Eric M. Yeatman, Alex J. Thompson

**Affiliations:** a Imperial College London, Institute of Global Health Innovation, The Hamlyn Centre, London, United Kingdom; b Imperial College London, Department of Electrical and Electronic Engineering, Faculty of Engineering, London, United Kingdom; c Imperial College London, Department of Surgery and Cancer, Faculty of Medicine, London, United Kingdom

**Keywords:** optical fiber, bacteria manipulation, plasmo-thermo-electrophoresis, plasmonics, drug delivery, two-photon polymerization

## Abstract

**Significance:**

Plasmo-thermo-electrophoresis (PTEP) involves using plasmonic microstructures to generate both a large-scale convection current and a near-field attraction force (thermo-electrophoresis). These effects facilitate the collective locomotion (i.e., swarming) of microscale particles in suspension, which can be utilized for numerous applications, such as particle/cell manipulation and targeted drug delivery. However, to date, PTEP for ensemble manipulation has not been well characterized, meaning its potential is yet to be realized.

**Aim:**

Our study aims to provide a characterization of PTEP on the motion and swarming effect of various particles and bacterial cells to allow rational design for bacteria-based microrobots and drug delivery applications.

**Approach:**

Plasmonic optical fibers (POFs) were fabricated using two-photon polymerization. The particle motion and swarming behavior near the tips of optical fibers were characterized by image-based particle tracking and analyzing the spatiotemporal concentration variation. These results were further correlated with the shape and surface charge of the particles defined by the zeta potential.

**Results:**

The PTEP demonstrated a drag force ranging from a few hundred fN to a few tens of pN using the POFs. Furthermore, bacteria with the greater (negative) zeta potential (|ζ|>10  mV) and smoother shape (e.g., *Klebsiella pneumoniae* and *Escherichia coli*) exhibited the greatest swarming behavior.

**Conclusions:**

The characterization of PTEP-based bacteria swarming behavior investigated in our study can help predict the expected swarming behavior of given particles/bacterial cells. As such, this may aid in realizing the potential of PTEP in the wide-ranging applications highlighted above.

## 1 Introduction

Micro/nanorobotics presents numerous opportunities in medicine ranging from microscale surgery to targeted drug delivery. Many approaches and techniques have been reported within the field of microrobotics, and some key examples include fuel-powered tubular microrockets,^1^ magnetically actuated helical swimmers,^2^ ultrasound-powered nanowire motors,^3^ and sperm-powered biohybrid microrobots.^4^ However, many challenges remain when applying microrobotics to medicine, including biocompatibility, retention, toxicity, and therapeutic efficacy.

Bacteria-based microrobots have been proposed due to the functional capabilities of bacteria to autonomously swim toward specific regions via chemotaxis (and other) mechanisms^5^ and to carry therapeutic payloads.^6^ Numerous external actuation mechanisms have been used to provide enhanced locomotion and targeting of bacteria for microrobotic applications. This includes magnetic and acoustic actuation as well as the use of optical tweezers.^7^^–^^13^ However, all of these approaches are limited in their ability to offer simultaneous control of large volumes of cells (i.e., control of bacterial “swarms”), which is vital for efficient use in drug delivery.

The phenomenon of plasmo-thermo-electrophoresis (PTEP) has been reported as an alternative technique for bacterial motion control. PTEP relies on the use of plasmonic metal microstructures to generate highly localized thermal gradients caused by surface plasmon resonance (SPR).^14^^–^^17^ The resulting plasmonic heating acts to induce both convection (mm-cm scale) and thermophoresis (motion of particles in response to a temperature gradient; μm scale).^18^ Thus PTEP can facilitate swarm generation and control. Importantly, PTEP does not require complex optics or high-powered illumination and can be deployed using relatively simple microstructures. Indeed, the use of PTEP for the manipulation of *E. coli* using custom-fabricated plasmonic optical fibers (POFs) demonstrating the potential for minimally invasive deployment was recently reported.^19^

Despite the potential opportunities offered by PTEP, it has yet to be fully characterized, and the prediction of the expected behavior (i.e., motion and forces) in different particles and cells has not been widely reported. As such, in this article, we present an investigation of PTEP in a series of microparticles and bacterial cells using POFs in combination with two optical imaging systems. Our results demonstrate that PTEP-induced motion is dependent on both particle shape and surface charge (zeta potential), with zeta potential providing an accurate prediction of motion/accumulation behavior.

## 2 PTEP Theoretical Background

The PTEP effect occurs when localized SPR is generated at metallic microstructures (which can be achieved by irradiating the microstructures with light of the appropriate wavelength). The SPR generates a highly localized heat source at the metallic microstructure, which has two critical effects. First, it generates a thermal convection current with a range of several hundred microns. This convective effect can be analyzed following Stokes law^20^ (assuming the fluid flow is laminar, i.e., Reynolds number ≪1). Assuming smooth, spherical particles and homogeneous material, when the Stokes number is below 1, objects within the flow will follow the streamlines (i.e., they will follow the flow direction and bypass any obstacles). When the Stokes number is >1, objects will fall out of the flow streamlines and collide with obstacles.

Second, in the near field, within a few tens of micron range,^21^ thermophoresis takes place to induce a micromigration of particles in suspension under a local heat source.^22^ This particle migration (particle flux, j) under an additional temperature gradient can be described by

$$j=-cD_{T}\nabla T-D\nabla c,$$ (1)

where c, $D_{T}$, ∇T, and D are the particle concentration (number of particles per unit volume), the thermophoretic mobility, the temperature gradient, and the diffusion coefficient derived from Fick’s law of diffusion (resulting from Brownian motion), respectively.^23^ The first term on the RHS of Eq. (1) describes the particle transport due to the temperature gradient, and the second term indicates the flux generated by the concentration gradient. The ratio between $D_{T}$ and D (Soret coefficient $S_{T}=D_{T}/D$) is defined to measure the direction and magnitude of thermophoresis. A negative $S_{T}$ indicates that the particles converge to the hot region, and a positive $S_{T}$ implies that particles will be pushed to the cold area. In practice, it is a highly sensitive and incomprehensible attribute affected by various factors, such as particle size,^22^ solvent properties, surface chemistry of the particles, and temperature.^24^ As an analogy with the other types of phoretic motion, such as electrophoresis, the particle drift velocity can be calculated by the following equation:

$$v_{D}=-D_{T}\nabla T.$$ (2)

Finally, in addition to the thermal effects, the electric field also needs to be considered as a factor affecting particle motion in PTEP. For particles with charged surfaces in an electrolyte solution (i.e., as in the experiments presented here), an external enhanced electric field can be induced by the plasmonic array due to the different thermal diffusivities of the ions in the solution. In turn, this can lead to further forces being exerted on the particles/cells due to the separation of electrical charge, which can further impact motion.^23^^,^^25^

In our study, we used POFs to achieve both convection and thermophoresis via plasmonic heating of the microstructure at the tip of the POF. In turn, this facilitated the manipulation of particle swarms using low optical powers based on a combination of the effects described above.

## 3 Methods

### 3.1 Fabrication of the Optical Fibers

**Fig. 1:**
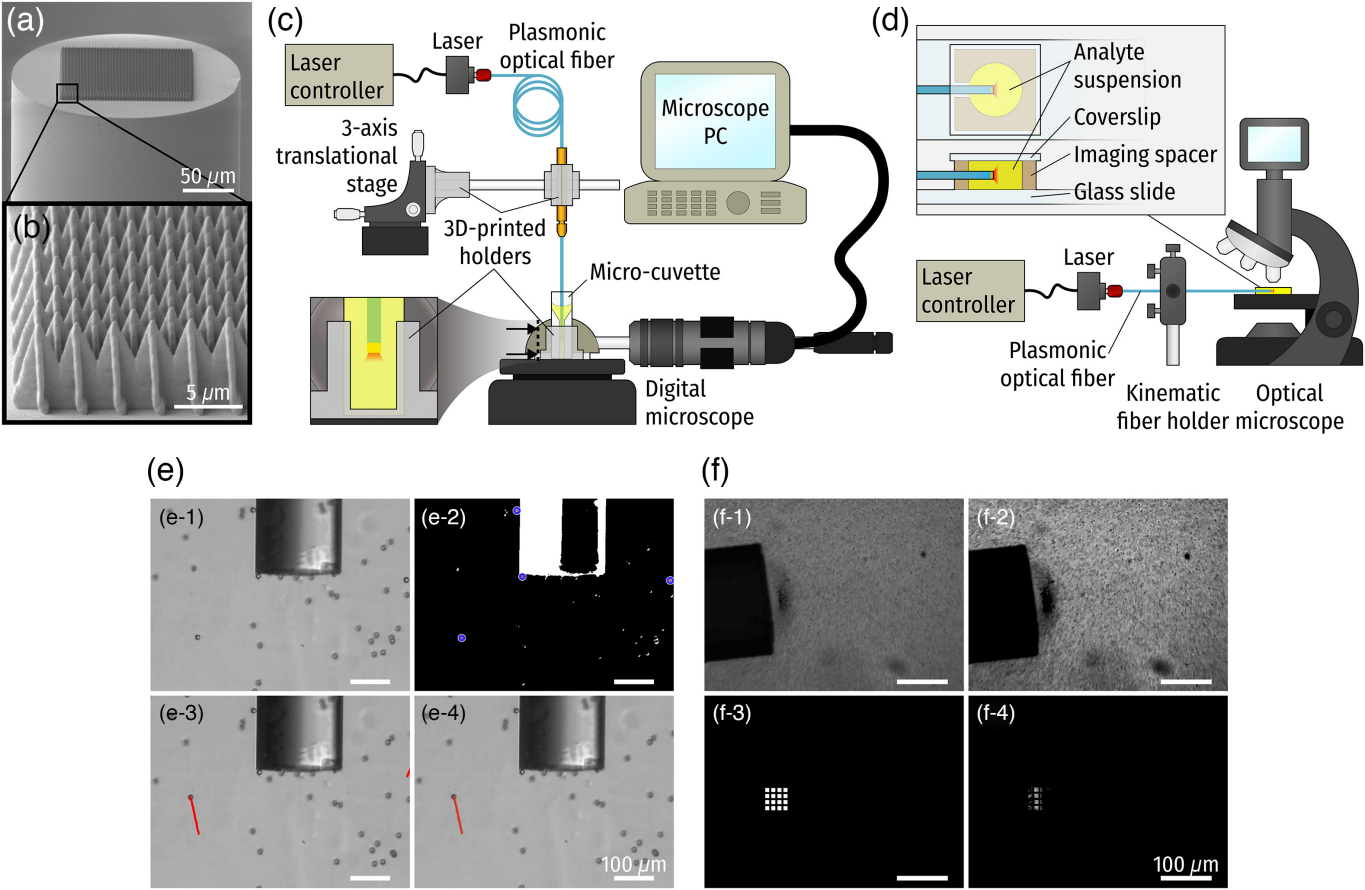
Experimental setup configurations and image analysis procedures. SEM images of (a) the plasmonic fiber (PF) and (b) the plasmonic microstructure array. (c) Vertical and (d) horizontal setup schematic. (e) PSs tracking in the vertical experimental setup: (e-1) conversion into a grayscale image; (e-2) binarization and particle detection; (e-3) particle trajectory tracing; and (e-4) out-of-frame particle rejection. (f) Procedures of quantifying the particle concentration change near the fiber tip: (f-1) Conversion into a grayscale image; (f-2) brightness/contrast adjustment; (f-3) 4×4 filter windows generation; and (f-4) pixel intensity measurement.

To investigate PTEP induced via POFs, three different optical fibers were prepared—bare fiber (BF), gold-coated fiber (GCF), and plasmonic fiber (PF), based on silica multi-mode optical fibers (200/220  μm core/cladding diameter, 0.22 NA, FG200LEA, Thorlabs, Inc., Germany). All fibers were cut to 96 mm long using an automatic optical fiber cleaver (CT-101, Fujikura Ltd., Japan). Gold thin film (50 nm) deposition for the GCF was carried out by sputtering (HEX thin film deposition system, Korvus Technology, United Kingdom). The PF [Fig. 1(a)] was fabricated by creating polymeric microstructure arrays using two-photon polymerization, followed by 50 nm gold thin film sputtering. Cross spike arrays [Fig. 1(b)], which were successfully validated to support effective plasmon excitation in previous works,^19^^,^^26^ were fabricated using the Photonic Professional GT system (Nanoscribe GmbH., Germany) and photoresist (IP-Dip, Nanoscribe GmbH., Germany).

### 3.2 Vertical Configuration Setup

One experimental setup was designed to mount the optical fibers vertically in a suspension of polystyrene microspheres (PSs) and to provide a view of PS behavior in suspension in the horizontal observing direction [Fig. 1(c)]. A fiber holder was designed and 3D printed to clamp the fiber while dipping it into the test suspension vertically. A three-axis translational stage connecting with the metal post by a 3D-printed right-angled optical post-holder was used to provide six degrees of freedom of the optical fiber. Additionally, a cuvette holder fixed to the sample stage of the microscope (VH-Z100R, Keyence Co., Japan) was 3D printed to secure stability and clear access from the side view. The proximal end of the fiber was connected to a laser diode (785 nm continuous wave, L785P090, Thorlabs, Inc., Germany) mounted within a laser diode mount with integrated temperature controller (LDM9T/M, Thorlabs, Inc., Germany), operated by a laser diode current controller (LDC202C, Thorlabs, Inc., Germany).

### 3.3 Horizontal Configuration

A horizontal experimental configuration allowed horizontal immersion of the optical fiber into suspension (10  μL) contained within an imaging spacer (thickness 360  μm). The imaging spacer accommodated the diameter of the optical fiber, and the proximal end was connected to a laser source. The imaging was taken by an upright optical microscope (LCD Digital Microscope II, Celestron, LLC, United States) with a 10× magnification lens (resulting in a pixel size of $0.89\times 0.89\;\mu m^{2}$). The schematic of the horizontal configuration is shown in Fig. 1(d).

### 3.4 Bacteria Sample Preparation

Five bacteria species were investigated in this work. *Escherichia coli* (EC) (ATCC BAA-769), *Staphylococcus epidermidis* (SEp) (ATCC 12228), *Staphylococcus aureus* (SAu) (ATCC 29313), and *Klebsiella pneumoniae* (KP) (ATCC 13883) were purchased from ATCC (United States) and *Streptococcus agalactiae* (SAg) (NCTC 8181) was purchased from NCTC (United Kingdom). Bacteria were aliquoted in glycerol stocks. They were cultured overnight in tryptic soy broth (Sigma-Aldrich, Germany) at 37°C with constant shaking (200 rpm). Afterward, the bacterial cultures were washed three times using phosphate-buffered saline (PBS). Finally, the precipitates (bacterial cells) were resuspended in fresh PBS using a vortex mixer.

### 3.5 Experiment

#### 3.5.1 Convection current measurement

To characterize the PS behavior, PS suspensions with three sizes, 1.1  μm (0.1% v/v), 5  μm (1% v/v), and 15  μm (1% v/v), in distilled water (DIW) were prepared. Their behavior under illumination through the optical fibers described above was monitored and investigated using the vertical experiment setup [Fig. 1(c)]. To examine the effect of the laser illumination power, the operating current of the laser was varied from 30 to 50 mA in 5 mA increments using the laser diode controller. In order to measure the exact output power at the distal ends of PFs, the distal ends were connected to the sensor area of a laser power meter (PM160, Thorlabs, Inc., United States) and the output power readings were recorded while controlling the current. The output power values were averaged from the measurements of two independent PFs.

#### 3.5.2 Bacteria accumulation effect

The behavior of the PSs and the selected five bacterial species (KP, EC, SAu, SEp, and SAg) was observed and analyzed using the horizontal experiment setup [Fig. 1(d)]. The concentration for the six samples was controlled to be roughly the same order of magnitude ($10^{7}\text{ cells}/\mathrm{mL}$). The laser power was maintained at a constant level (with a laser diode operating current of 75 mA). In addition, a comparison between bacteria (KP) suspended in DIW and PBS was also performed to examine the effect of the solvent polarity. All videos were recorded for 8 min, and the frame rate was 30 frames per second.

### 3.6 Image Segmentation (PSs)

**Fig. 2:**
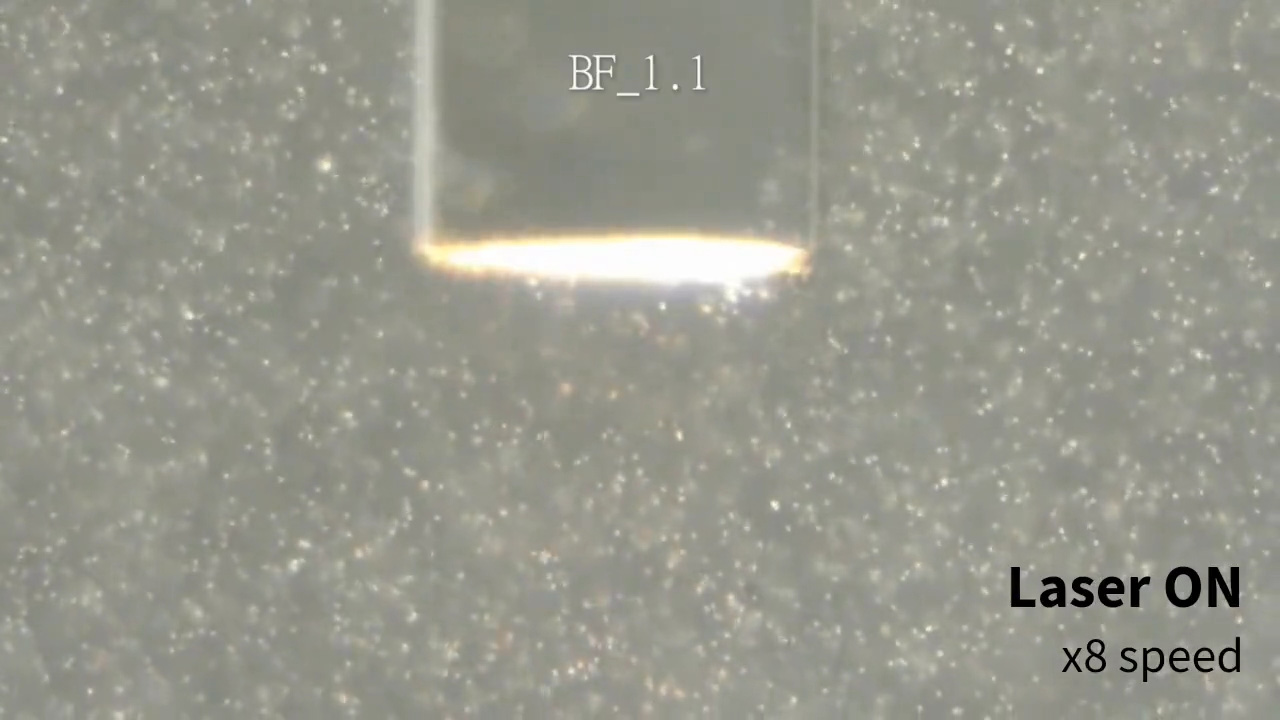
1.1  μm PS particles motion under laser irradiation through different optical fibers. BF, bare fiber; GCF, gold-coated fiber; PF, plasmonic fiber (Video 1, mp4, 10 MB [URL: https://doi.org/10.1117/1.JBO.28.7.075003.s1]).

**Fig. 3:**
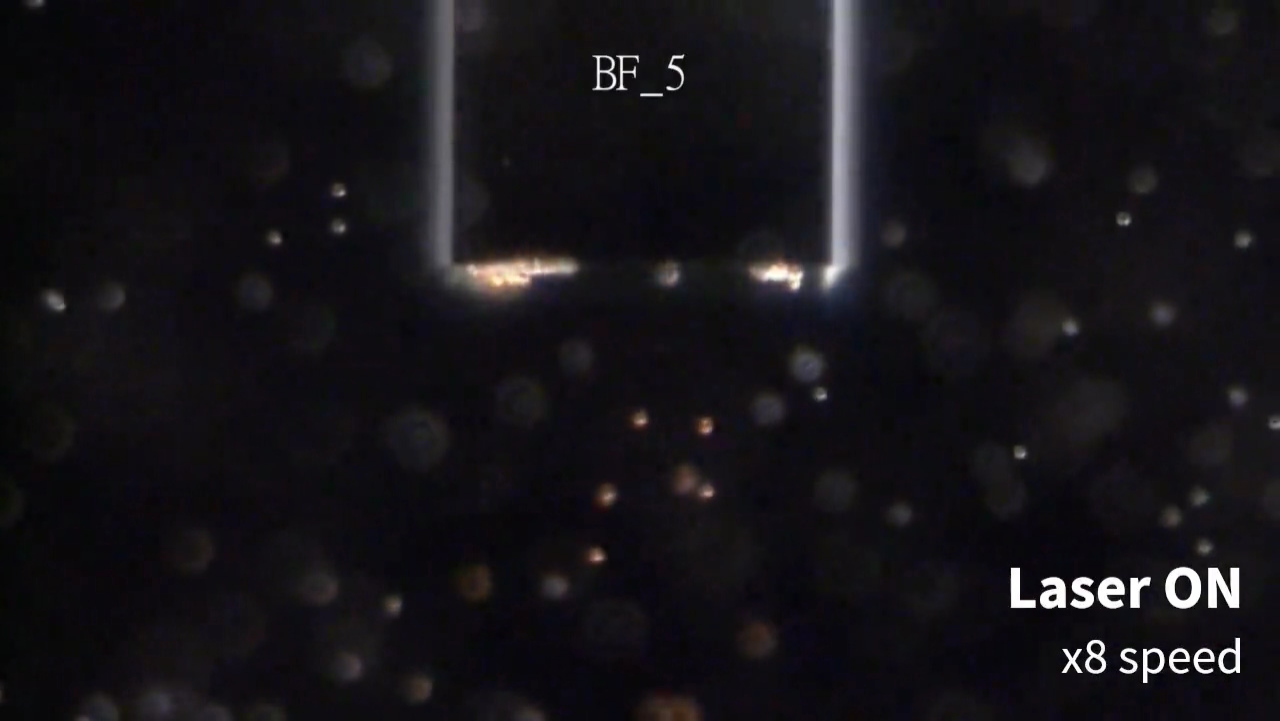
5  μm PS particles motion under laser irradiation through different optical fibers. BF, bare fiber; GCF, gold-coated fiber; and PF, plasmonic fiber (Video 2, mp4, 9.95 MB [URL: https://doi.org/10.1117/1.JBO.28.7.075003.s2]).

**Fig. 4:**
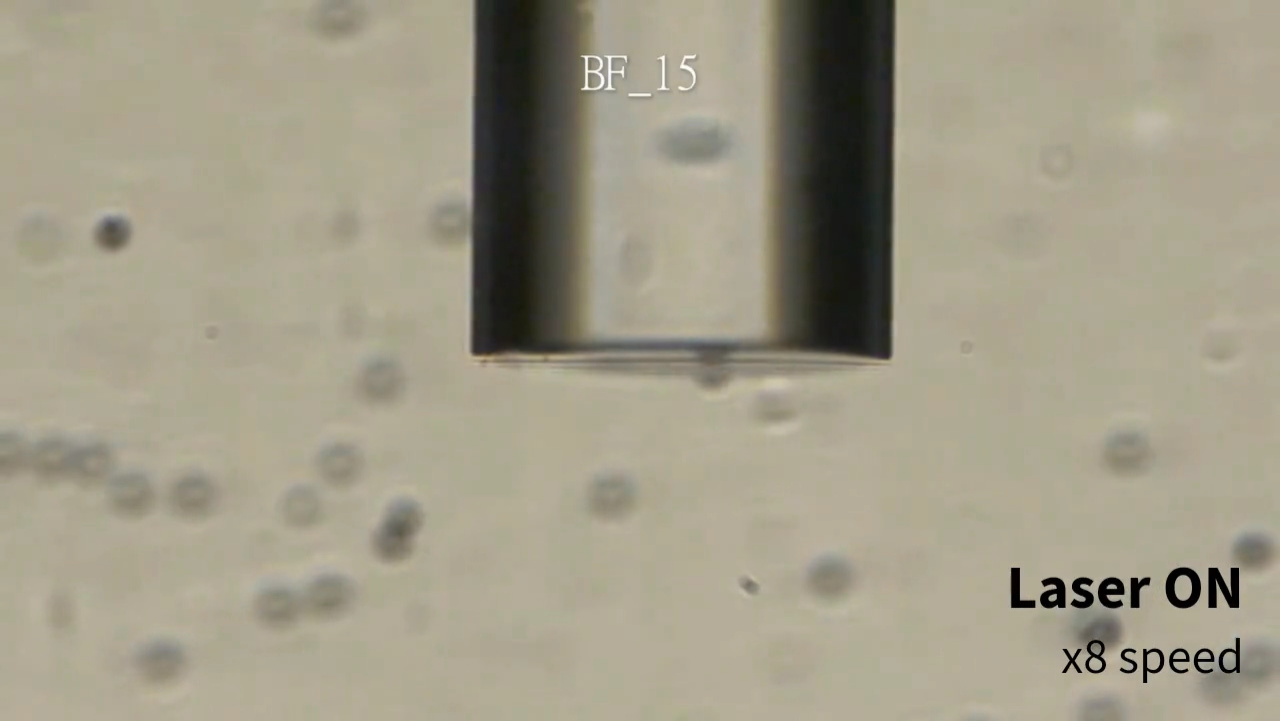
15  μm PS particles motion under laser irradiation through different optical fibers. BF, bare fiber; GCF, gold-coated fiber; PF, plasmonic fiber (Video 3, mp4, 9.88 MB [URL: https://doi.org/10.1117/1.JBO.28.7.075003.s3]).

To characterize the behavior of PSs in suspension using three different fibers, images from the recorded videos (28 frames per second, Figs. 2–4) were processed using MATLAB. To obtain particle velocity and other relevant parameters, the positions of the PSs must first be detected in each frame. The center points of the PSs in each image frame were found using the circular Hough transform algorithm (which detects circle patterns and returns the center points and radii). This approach worked well for the PS experiments due to the spherical shape of the PSs.

Having detected the PS positions in each image frame, the first frame was set as the reference frame and tracked PS trajectories were then defined relative to their initial position in the reference frame. The trajectories for individual PSs were determined by finding the closest position in the next frame to the detected position in the previous frame. After the trajectories for all PSs were recorded, the lengths of the trajectories were filtered to remove PSs that were not in the focal plane for the duration of the experiment [Fig. 1(e)].

After obtaining the position data of the PS trajectories, particle velocities were calculated according to the change in position and the time between frames. The Reynolds number was calculated based on the velocity to confirm whether the PS behavior satisfied Stokes’ law. The Stokes number was then used to verify the PS motion mode theoretically. Average velocities (across entire trajectories) were also calculated to compare velocities for different PS sizes and laser excitation powers.

### 3.7 Quantification of the Concentration Change

To assess the accumulation of bacteria and PSs near the fiber tip, particle concentrations were determined by measuring the changes in image pixel intensity in selected areas. The greater the number of bacteria and PSs concentrate in the vicinity of the fiber tip (particle concentration increase), the darker the image gets (pixel intensity decreases) because the particles block or scatter the transmission illumination. Prior to analysis, all image frames were converted to grayscale (i.e., 0 to 255) and then normalized to provide a constant brightness and contrast across all frames. To assess the concentration of particles/cells at the fiber tip, we defined 16 “filter windows” (10×10  pixels per window) in a 4×4 grid in the region in which particle accumulation was expected (i.e., at the fiber tip).

The concentration of particles or bacterial cells (C) was calculated by the highest pixel intensity (255) minus the average pixel intensity (I) in each filter window (i.e., C=255−I). The concentration versus time (C−t) profiles from all filter windows were then plotted to facilitate analysis and interpretation of accumulation behavior. To reduce the noise of the C−t curves, a moving average window (size of 300=10.7  s) was applied. Figure 1(f) illustrates the procedure of quantifying concentration changes. Finally, the accumulation ability for the bacteria and the 1.1  μm PSs was quantified based on both their accumulation speed (i.e., time to reach saturation) and final accumulation area.

**Fig. 5:**
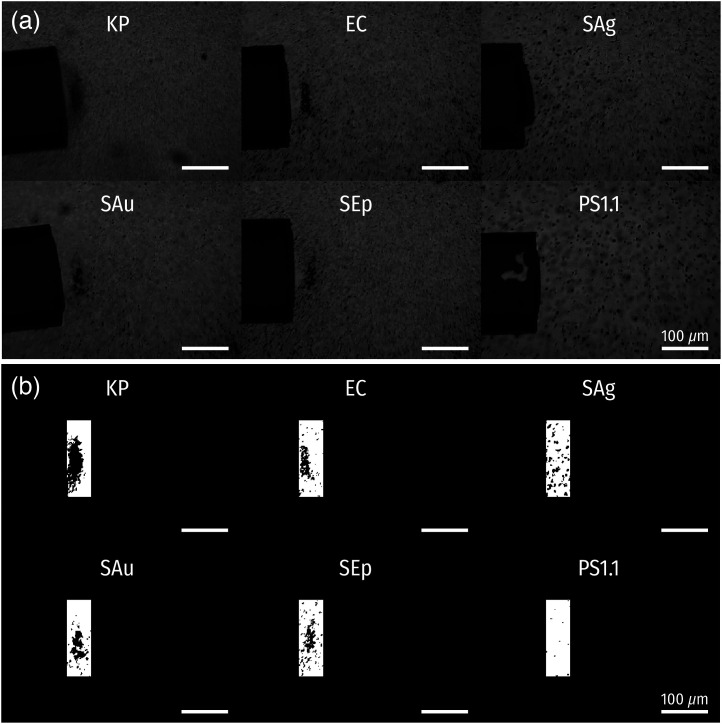
Image processing for characterization of particle accumulation. (a) Example images of accumulating particles following grayscale conversion and normalization. (b) Rectangular ROIs used for calculation of accumulation areas.

The accumulation area was measured from the last frame of the laser-on period (t=192  s), where the swarm size was saturated [Fig. 5(a)]. The images were binarized to facilitate the segmentation of the swarm areas from the background [Fig. 5(b)]. Subsequently, rectangular region of interest (ROI) windows were set to filter the binarized images. The positions of the ROI windows were adjusted with respect to the positions of the optical fibers in each image to cover the swarms observed in each experiment [Fig. 5(b)]. Finally, the dark pixels within the ROI windows were counted to obtain the size of the accumulation area in each experiment.

The accumulation speed was extracted from the C−t profiles. As the particles were expected to start to accumulate once the laser was turned on, the initial slope of the concentration curve was defined as the accumulation speed. The accumulation speed values of the first 10 s were used to calculate the slope of the curve.

### 3.8 Characterization of Bacteria Accumulation

We examined particle shape and zeta potential to determine the cause of the different accumulation behaviors. The influence of the shape of bacterial cells was investigated via microscopic imaging [scanning electronic microscope (SEM), LYRA3 FIB-SEM, TESCAN, Czechia; Fig. S1 in the Supplementary Material]. The zeta potential of all particles was measured using a zeta potential analyzer (Zetasizer Nano ZSP, Malvern Panalytical, United Kingdom).

## 4 Results and Discussion

### 4.1 PS Behavior Under Plasmonic Heating

**Fig. 6:**
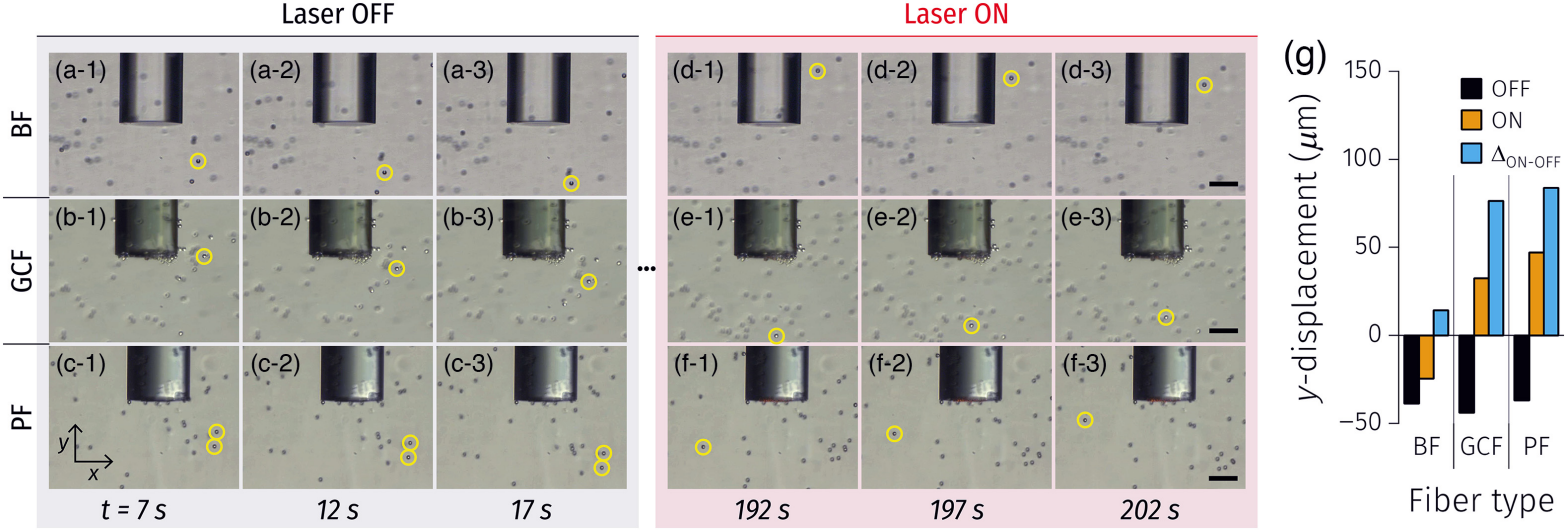
(a)–(f) PSs particle tracking under laser irradiation through various optical fibers. BF, bare fiber; GCF, gold-coated fiber; and PF, plasmonic fiber. $d_{p}=15\;\mu m$, Scale bars: 100  μm. (g) Average y-displacements before and after laser irradiation over 5 s of time intervals of tracked particles in the images (a)–(f) and their differences.

The motion of 15  μm PSs under illumination through the three types of optical fibers (i.e., BF, GCF, and PF) was analyzed to characterize the plasmonic heating of the optical fibers and resulting particle behavior (Fig. 6). Since the measurements were conducted using the vertical measurement setup [Fig. 1(c)], there was a gravitational force acting (−y direction in the images). Therefore, PS motion in the −y direction was observed for all fibers when there was no laser illumination [Figs. 6(a)–6(c)]. However, when the laser was on, different PS behaviors were observed [Figs. 6(d)–6(f)]. While no significant change in particle motion was observed with laser illumination through the BF, an apparent directional change was detected in experiments with the GCF and PF. This indicates that additional external force(s) were generated against the gravitational force (more accurately, the differential net force between gravity and buoyancy). The 50 nm gold thin film coated onto the GCF was sputtered, and it is well known that sputtered gold contains relatively smaller and larger grains resulting in nanoscale roughness. This can support plasmon excitation on the gold surface (and, hence, plasmonic heating), creating a local temperature gradient and thus generating convection currents. Notably, the y-displacement change of the particles under the PF was more significant than that under the GCF [Fig. 6(g)], indicating a more substantial plasmonic heating effect by the PF. This is attributed to the higher plasmon excitation efficiency with the extra microscopic features of the plasmonic structures at the fiber tips.^19^

### 4.2 Particle Motion Analysis

**Fig. 7:**
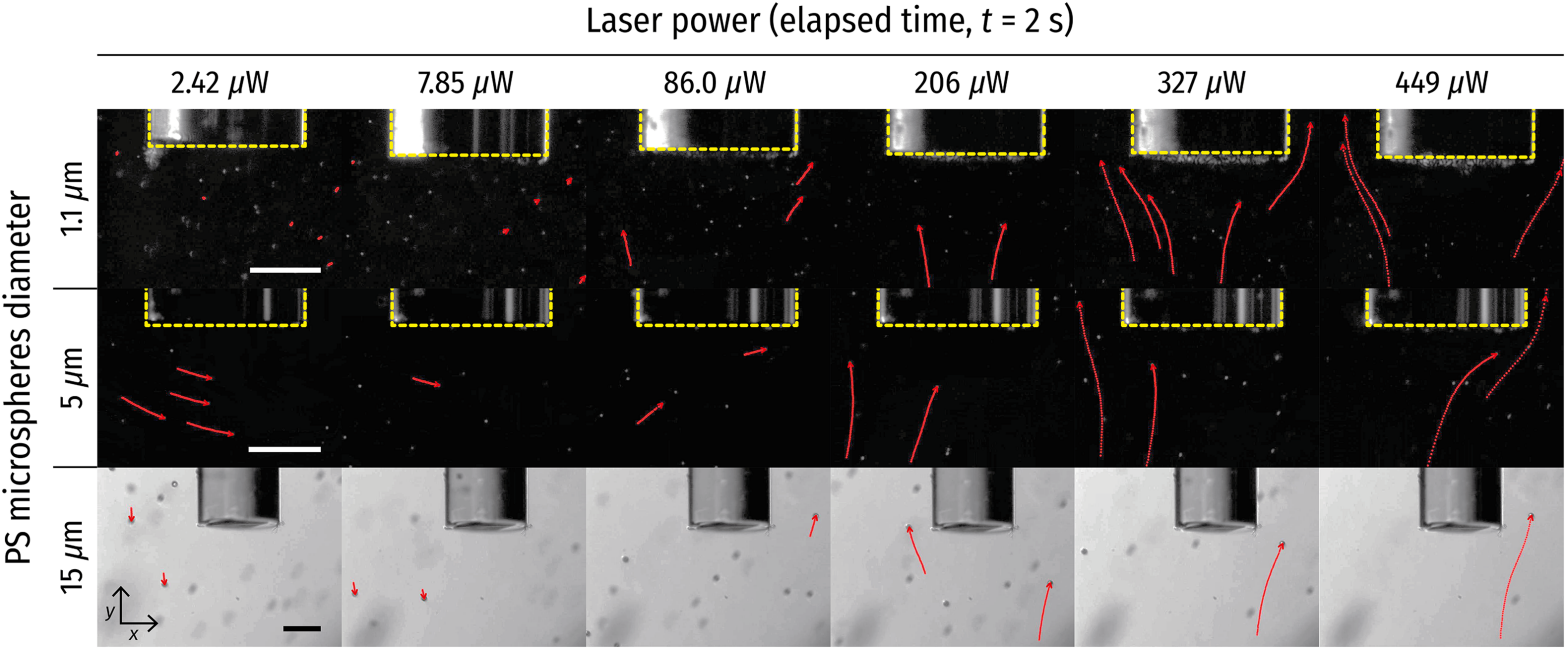
Extracted frames of the video data of 1.1/5/15  μm PSs under varying laser power at t=2  s. The red lines indicate the tracked PSs trajectories. Scale bars: 100  μm.

**Fig. 8:**
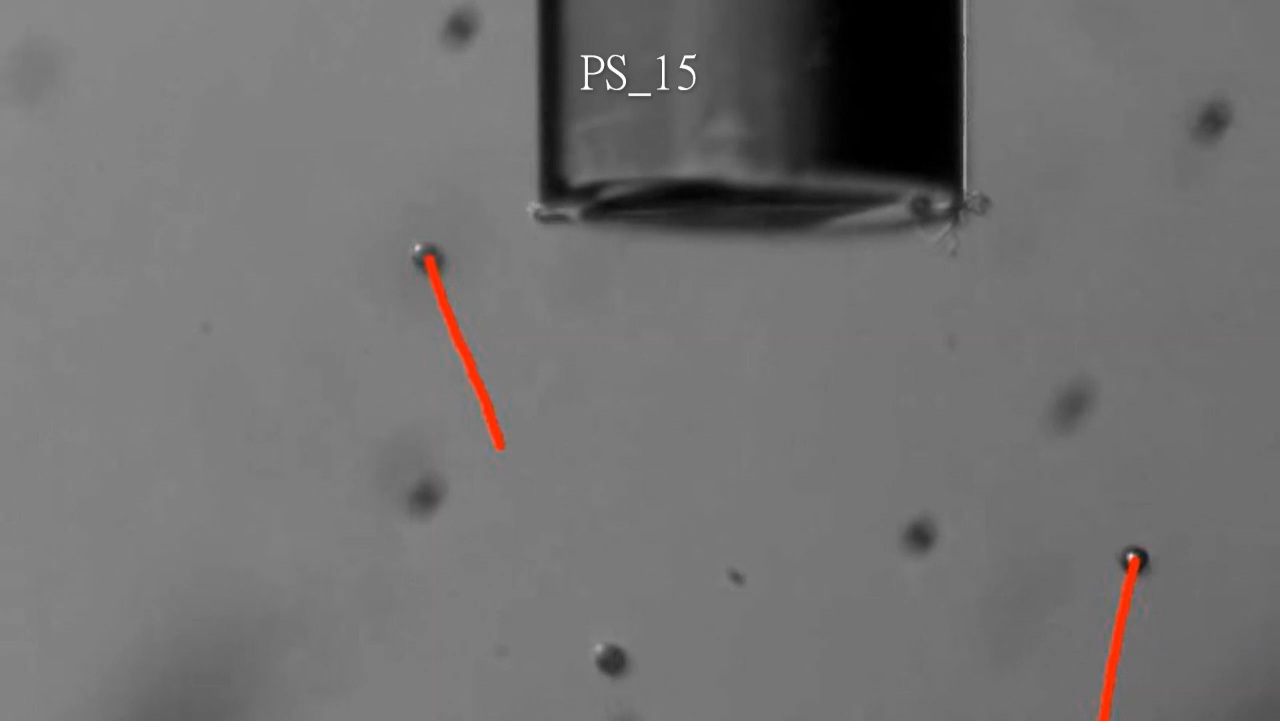
Representative visualizations of PS particle (1.1, 5, and 15  μm) tracking under 206  μW laser irradiation via PFs. (Video 4, mp4, 3.19 MB [URL: https://doi.org/10.1117/1.JBO.28.7.075003.s4]).

**Fig. 9:**
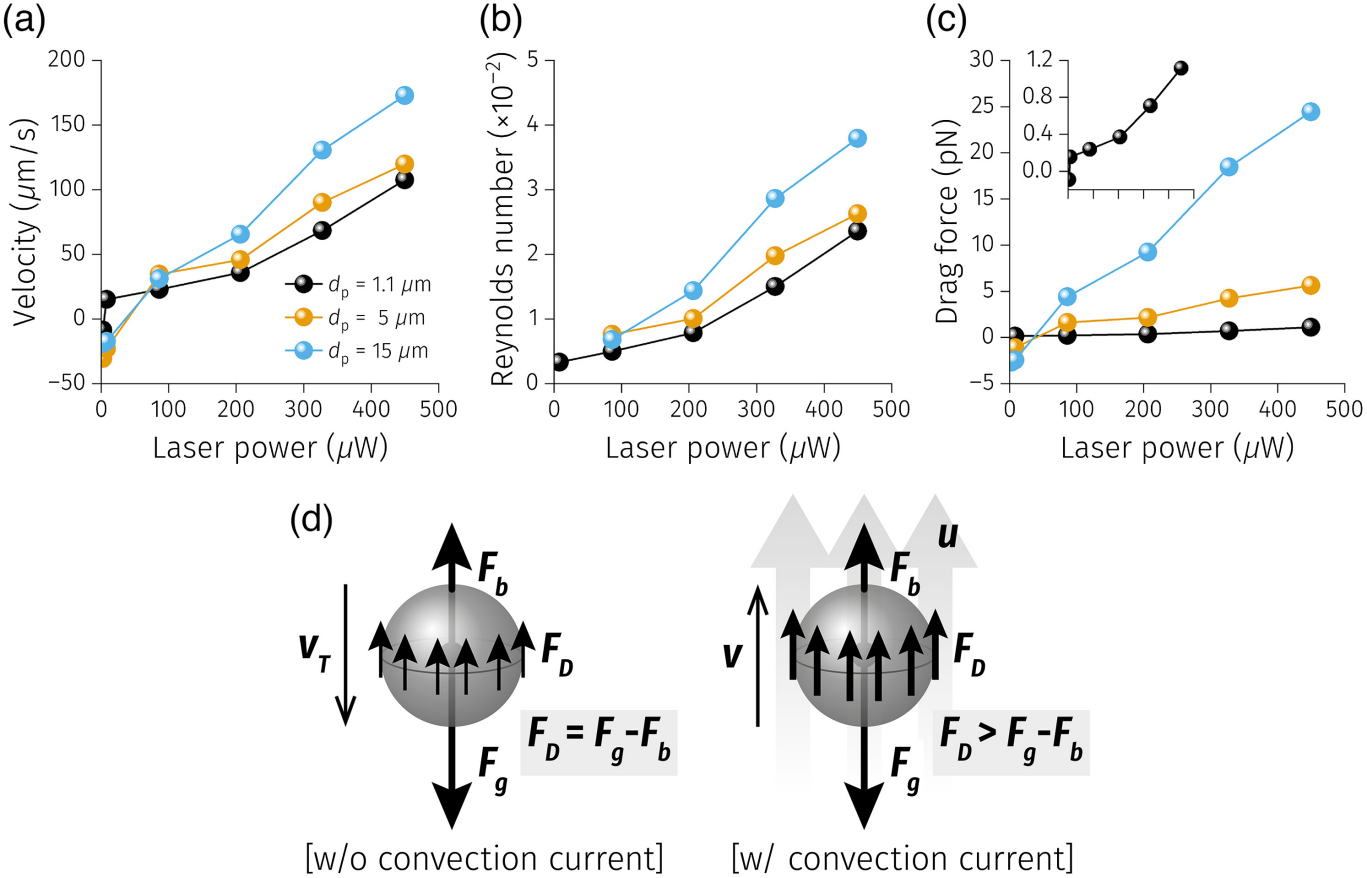
Relevant mechanical properties of PSs under plasmonic heating. (a) PSs velocity, (b) Reynolds number, and (c) Stokes drag force. (d) The force and motion diagrams of PSs experiencing a gravitational force ($F_{g}$), buoyancy ($F_{b}$), and the Stokes drag force ($F_{D}$) without and with the convection current caused by plasmonic heating.

Next, PSs behavior under laser irradiation through the PF with various laser powers was characterized to analyze the relationship between the plasmonic heating and the resulting force on the PSs due to the generated convection current (Figs. 7 and 8). The laser diode current was varied from 30 to 50 mA in 5 mA increments, resulting in 2.42, 86.0, 206, 327, and 449  μW in measured irradiation power at the distal end of the PF. Additionally, 31.8 mA (yielding an irradiation power of 7.85  μW) was included because it was observed that 1.1  μm PSs started to move upward (+y direction) at that value. The larger particles exhibited upward motion under the laser irradiation power of 86.0  μW. Furthermore, the particle travel distance increased over the same elapsed time with increased laser power. It is important to note that the laser power drastically jumped under the electric current between 31.8 and 35 mA, as this range contained the lasing threshold. Thus the exact threshold laser power in which the upward force was in equilibrium with the gravitational force was not directly measurable for the larger PSs (5 and 15  μm). However, the dataset was informative enough to derive the mechanical properties of the particles to understand the PSs behavior under plasmonic heating (Fig. 9).

First of all, the velocity of particles was calculated from the particle travel distance (Δs) divided by the time interval (Δt=1/frame rate=1/(30  fps)=0.033  s) over consecutive frames. The velocities of particles were then averaged along the whole trajectory and the number of tracked particles [Fig. 9(a)]. The particle velocity was approximately linearly proportional to the laser power for all PSs. Furthermore, the larger the particle size, the higher the velocity that was achieved. This can be explained by the fact that the upward drag force on the particle is proportional to the cross-sectional area perpendicular to the fluid flow. To further investigate the particle motion, the Reynolds number and Stokes number were also calculated.

The Reynolds number (Re, the ratio of inertial forces to viscous forces within a fluid) was calculated to investigate the flow regime around the optical fibers. It is defined as Re=ρfuL/μ, where $\rho_{f}$ is the density of the fluid ($\rho_{f}=997\;\mathrm{kg}/m^{3}$ for DIW), u is the fluid speed, L is a characteristic length (optical fiber diameter in this study, L=220  μm), and μ is the fluid dynamic viscosity ($\mu =10^{-3}\;\mathrm{Pa}\cdot s$ for DIW). Here the tracked particle velocity was used as the fluid speed (u) because the particle motion was instantaneous with the laser irradiation and constant over time, representing the fluid flow adequately (Fig. S2–S4 in the Supplementary Material). Figure 9(b) displays the Re for each size of PS with respect to the varying laser power. Re≪1 in all cases indicates that the fluid in the system behaved as a laminar flow. Together with this laminar flow condition since the PSs are spherical, the particle behavior was assumed to meet Stokes’ law.

According to Stokes’ law, the Stokes drag force is defined as $F_{D}=-3\pi \mu d_{p}v$, where $d_{p}$ and v are the diameter and the velocity of PSs, respectively. The calculated drag forces applied on the PSs with different particle diameters under varying laser irradiation power are displayed in Fig. 9(c). As mentioned above, PSs experience a gravitational force ($F_{g}$), buoyancy force ($F_{b}$), and drag force [Fig. 9(d)]. In the case of the absence of the convection current due to plasmonic heating, the net force between $F_{g}$ and $F_{b}$ ($F_{g}-F_{b}$) is in equilibrium with $F_{D}$, resulting in particles traveling downward (due to gravity) at their terminal velocity, $v_{T}$ [Fig. 9(d) left]. However, when plasmonic heating starts creating the convection current, $F_{D}$ becomes larger and exceeds the net force between $F_{g}$ and $F_{b}$, leading to upward motion [Fig. 9(d) right]. As $F_{g}$ and $F_{b}$ exerting on the PSs ranging from a few fN ($d_{p}=1.1\;\mu m$) to a few tens of pN ($d_{p}=15\;\mu m$), it is rationally derived that $F_{D}$ values (which were observed to push the particles upward) range from a few hundred fN to a few tens of pN.

### 4.3 PTEP Accumulation of Bacteria

**Fig. 10:**
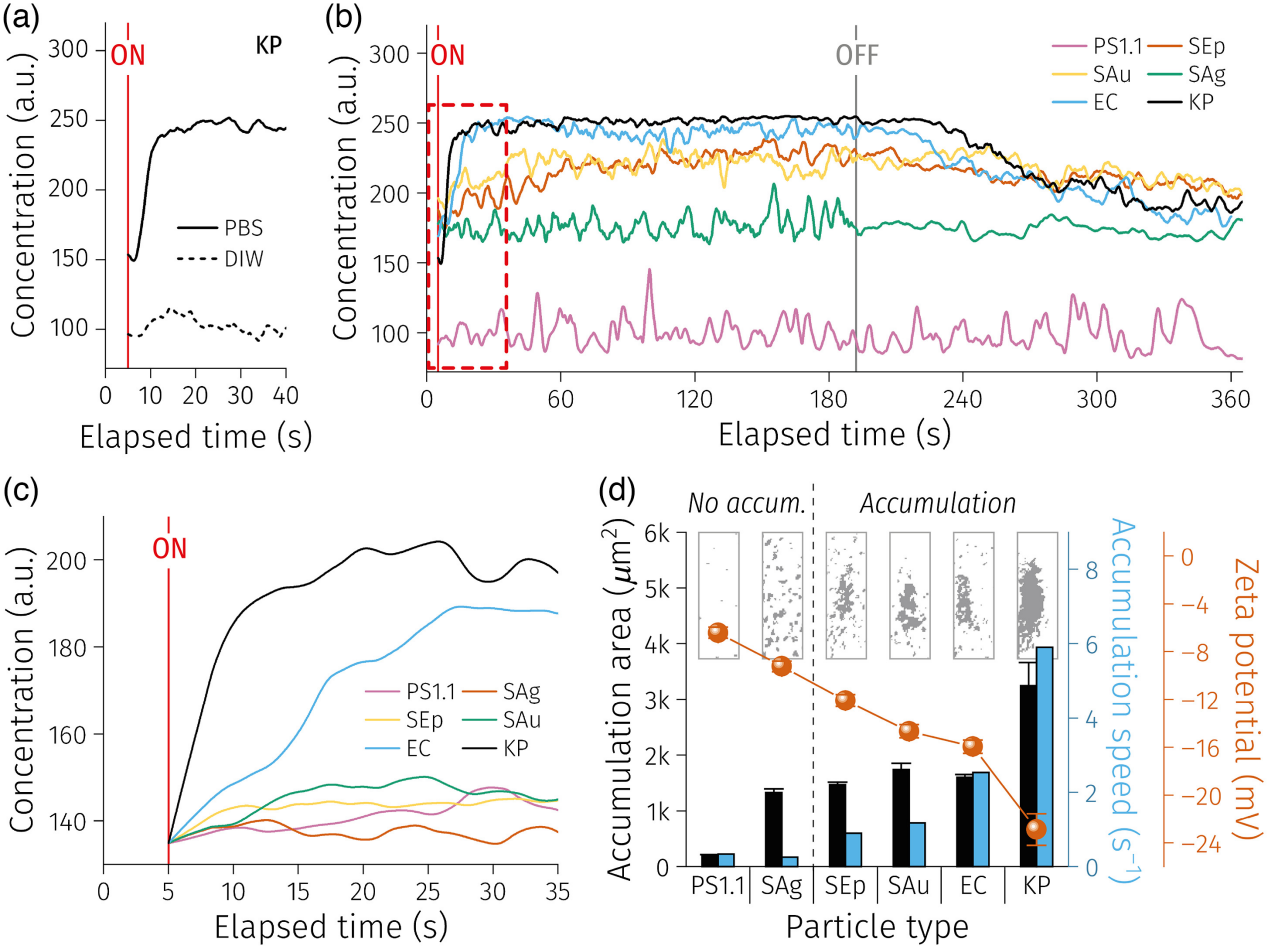
(a) Comparison between KP in DIW and PBS. (b) Overall concentration changing results of seven samples. (c) Smoothed profiles [“robust quadratic regression (rlowess)” smoothing window, filter size = 200 (7.1 s)] during the early time period for extraction of the accumulation speed values. (d) Summary results of accumulation area, speed, and zeta potential for the analyte suspensions. Inset: binary images of particle accumulations at t=192  s.

Although the plasmonic heating and the resulting local temperature gradient were successfully demonstrated to be effective in generating convection currents (resulting in far-field particle motion toward the PFs), particle accumulation at the fiber tip was not apparent in suspensions in DIW. This is where the PTEP effect ought to take place in order to facilitate local particle accumulation with the help of the interactions between the particle surface charge and the surrounding ions in the medium. Indeed, it was evident that KP suspension in PBS exhibited accumulation while those in DIW did not [Fig. 10(a)]. Therefore, PBS was chosen as the default medium for bacteria accumulation experiments.

**Fig. 11:**
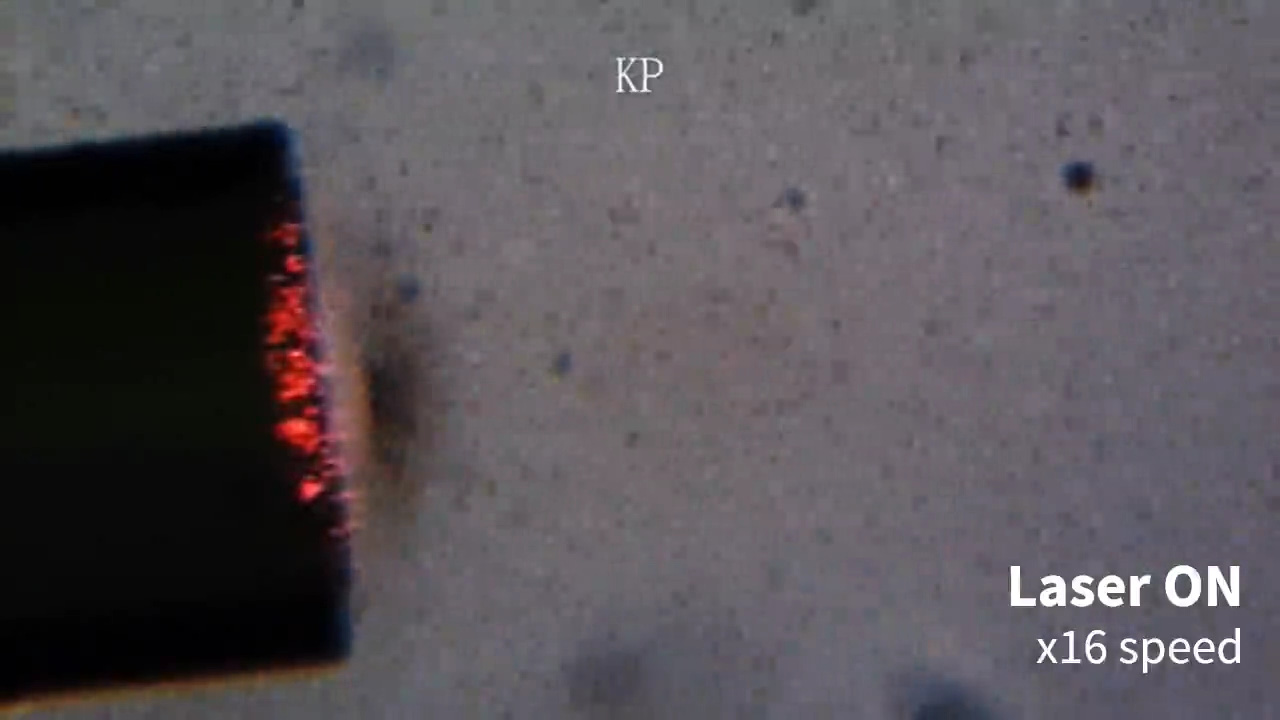
Accumulation behavior of bacterial/synthetic particle suspensions (KP, EC, SAg, SAu, SEp, and PS1.1) at the tip of PF (Video 5, mp4, 9.16 MB [URL: https://doi.org/10.1117/1.JBO.28.7.075003.s5]).

Figure 10(b) displays the concentration–elapsed time (C−t) profiles for five different bacterial species alongside the C−t profile for the 1.1  μm PSs (PS1.1) (Fig. 11). KP demonstrated the most substantial accumulation ability (i.e., concentration increase) followed by EC, and SAu and SEp showed relatively low accumulation ability. Interestingly, almost negligible accumulation was observed from SAg and PS1.1. Recalling the observation that PS1.1 experienced a drag force of $F_{D}>1.2\;\mathrm{pN}$ at the laser power over 449  μW [Fig. 9(c)], it can be deduced that the force attracting the PS1.1 particles toward the fiber tip was weaker than 1.2 pN in this case. To further quantify the accumulation ability of the particles, we analyzed the size of the bacterial swarms [accumulation area = the number of dark pixels × pixel size ($0.89\times 0.89\;\mu m^{2}$)] and the accumulation speed (concentration increase divided by the time until the saturation in $s^{-1}$) extracted from the C−t profiles [Figs. 10(c) and 10(d)]. The accumulation area and speed trends follow each other—increasing with the particles demonstrating stronger accumulation. It should be noted that SAg tended to form scattered clusters near the PF tips, but no recognizable single swarm as the indication of an effective accumulation [Fig. 10(d) inset]. Therefore, even though the accumulation area values of SAg and SEp were comparable (because the total number of dark pixels are similar), it needs to be carefully interpreted that SAg did not produce apparent accumulation, whereas SEp did.

To explain the particle behavior in the PTEP environment, the electrophoretic mobility also needs to be investigated (even though the temperature gradient effect is also important). In an electric field, the electrophoretic mobility for charged particles relies on the ion composition of the media, zeta potential (i.e., surface charge), and the shape of the particles.^27^^–^^30^ Accordingly, the difference in the electric double layer (EDL) can be regarded as an entry point to analyze the accumulation ability. Hence, it is essential to measure the zeta potential of the particles to understand their accumulation behaviors better.

As such, the zeta potentials of the bacteria cells and PS1.1 were measured and co-plotted in Fig. 10(d). All the particles were verified to have negatively charged surfaces ranging between −4 to −24  mV, and greater (negative) surface charge values were correlated with stronger accumulation ability.

Bacteria typically have a net negative electrostatic charge due to the chemical composition of the cell wall.^31^ In general, lipopolysaccharide and teichoic acid on the outermost layer of Gram-negative (e.g., EC and KP) and Gram-positive (e.g., SAg, SEp, and SAu) bacteria, respectively, contribute to the net negative surface charge. In addition, it was reported that Gram-negative bacteria possess more negatively charged surfaces than Gram-positive bacteria,^32^^,^^33^ which supports our observations.

**Fig. 12:**
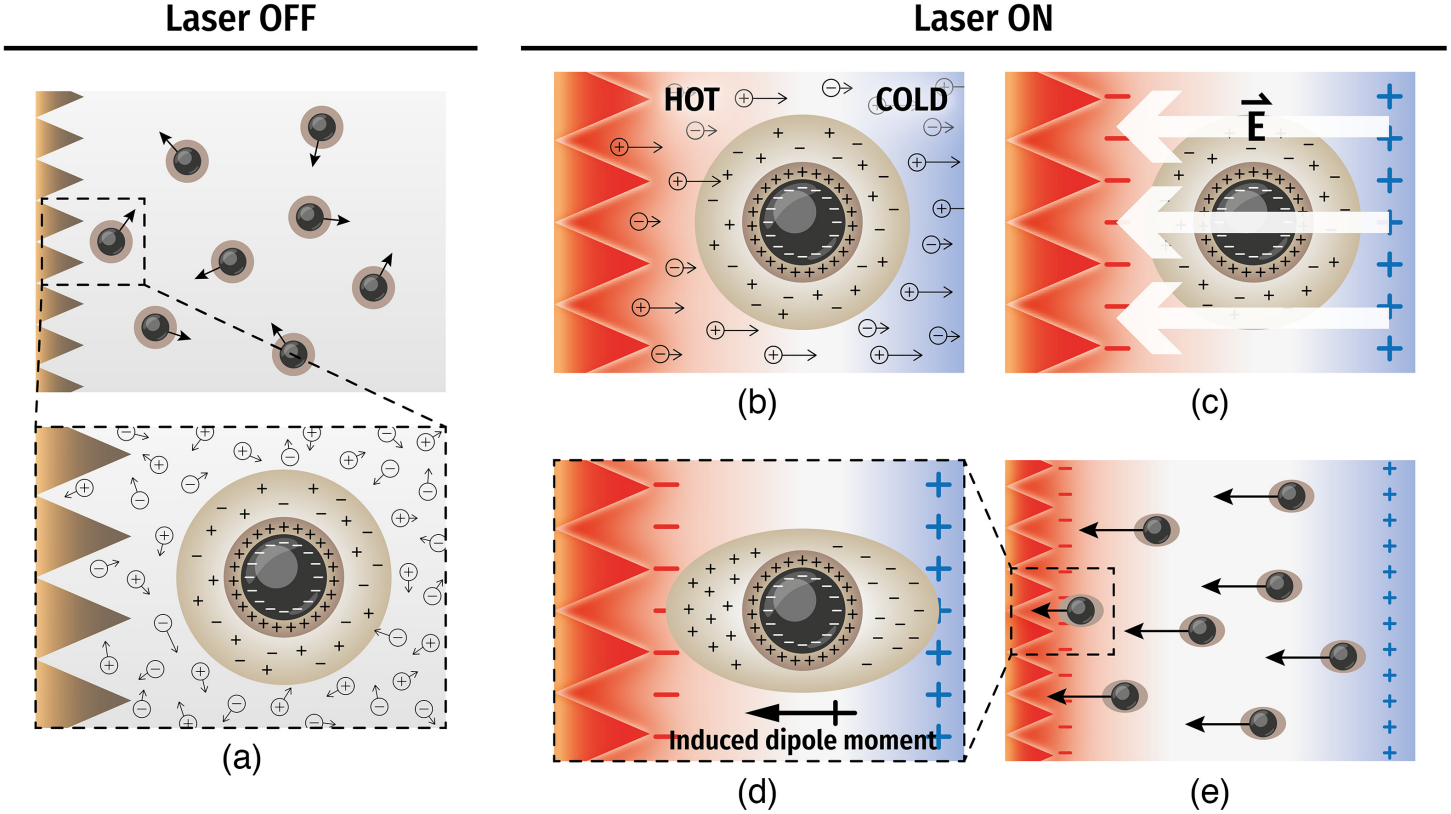
Schematic illustration of particle motion under PTEP effect near the tip of PF. (a) Particles suspended in electrolyte solution (Brownian motion); (b) differential ionic thermophoresis (Seebeck effect); (c) local electric field generation; (d) induced polarization of the double layer; and (e) induced dipole attraction toward the plasmonic tip.

Moreover, the ion concentration in the medium is another crucial variable in PTEP because it determines the direction of the thermoelectric field.^23^ Different ions have different Soret coefficients ($S_{T}$), and thus there can be a local electric field formed by the separation of the ions at the vicinity of the PF tip (Fig. 12). The $S_{T}$ of the ions comprising PBS can be found in the literature.^34^^,^^35^ Since 95% of the ion composition of PBS is ${\mathrm{Na}}^{+}$ and ${\mathrm{Cl}}^{-}$, the system can be approximated as a medium containing only these two ions. $S_{T}$ of ${\mathrm{Na}}^{+}$ and ${\mathrm{Cl}}^{-}$ are $4.69\times 10^{-3}$ and $7.18\times 10^{-4}\;K^{-1}$, respectively, meaning that the cation (${\mathrm{Na}}^{+}$) is more responsive to the temperature gradient. This disparity in S_T_ of these ions [“Seebeck effect,” Fig. 12(b)] eventually leads to an ionic concentration gradient, resulting in a local electric field generation [Fig. 12(c)]. Finally, this local electric field generates an induced dipole on a particle at the near field and promotes electrostatic attraction to create accumulation [Figs. 12(d) and 12(e)].

Another factor that could play a role in PTEP accumulation is the shape of the particles. Drag force is proportional to the drag coefficient $C_{D}$ associated with the particle shape.^36^ The drag coefficient of particles with a shape with more indentations is larger than particles with smooth surfaces. The rod-shaped bacteria (KP and EC) are smoother than connected spherical shapes (SAu and SEp) and chain shapes (SAg). The chain shape contains the most indentations over the surface, and thus the drag force exerted on SAg can potentially exceed the PTEP attraction force, towing the bacteria along the convection current streamline, eventually resulting in poor accumulation at the PF tips.

The overall results, together with the properties of the particles investigated in this work, are summarized in Table 1. Notably, while the rod-shaped Gram-negative bacteria (EC and KP) showed effective accumulation ability, the round-shape particles, including Gram-positive bacteria (SAg, SEp, and SAu) and PSs, either did not show noticeable accumulation (PS1.1 and SAg) or exhibited very weak accumulation performance (SEp and SAu). This is attributable to the interplay between the particle surface charge (zeta potential) and the particle shape. The motility of particles did not seem to contribute to the effect. Interestingly, as we observed strong accumulation for Gram-negative bacteria and weak (or no) accumulation for Gram-positive bacteria, these results may also suggest that the PTEP effect can be used to aid bacterial detection and classification (alongside the original target application for bacterial manipulation).

**Table 1:** Summary of particle properties and the PTEP accumulation performances.

Particle	Accum?	Particle type	Motility	Size (μm)	Shape	ζ (mV)	$S_{\text{accum}}$ ($s^{-1}$)	$A_{\text{accum}}$ ($\mu m^{2}$)
PS1.1	No	Synthetic microspheres	Non-motile	1.1	Sphere	−6.43	0.33	210
SAg	No	Gram(+) bacteria	Non-motile	0.6 to 12	Chain	−9.24	0.25	1328
SEp	Weak	Gram(+) bacteria	Non-motile	0.5 to 1	Round (cocci)	−12.1	0.90	1472
SAu	Weak	Gram(+) bacteria	Non-motile	0.5 to 1.5	Round (cocci)	−14.7	1.18	1739
EC	Strong	Gram(−) bacteria	Motile	0.5 to 2 × 2 to 6	Rod	−15.9	2.53	1605
KP	Strong	Gram(−) bacteria	Non-motile	0.5 × 2	Rod	−22.9	5.90	3250

ζ, particle zeta potential; $S_{\text{accum}}$, accumulation speed; and $A_{\text{accum}}$, accumulation area.

## 5 Conclusion

In this paper, we investigated the PTEP effect in PSs and five bacterial species with the aim of identifying the key underlying factors that determine the behavior of given particles under PTEP. The convection current, as the far-field driving force to pull the particles toward the plasmonic fiber tips, and the near-field thermoelectric effect to drag and accumulate the particles/cells, were both examined. On the basis of the investigation of particle accumulation capacity in an electrolyte solution, both particle shape and zeta potential were identified as key factors influencing accumulation ability. In particular, particles with a higher absolute zeta potential value will exhibit stronger accumulation ability. Furthermore, our observation of stronger accumulation ability in Gram-negative bacteria (relative to Gram-positive) can potentially offer additional opportunities for the use of the PTEP effect in the detection and classification of bacteria. Overall, these findings can help to guide the choice of bacterial species for use in bacteria-based microrobotic experiments, for example, for further investigation of bacteria-based targeted drug delivery systems. In the future, further scientific work will focus on enhancing the trapping/manipulation effects of PTEP and demonstrating the capabilities of this phenomenon for applications in microrobotics and drug delivery.

## Code, Data, and Materials Availability

This work includes video analysis MATLAB codes available on request. Supplementary Materials are available online.
