# Supplementary material for: Characterization of bacteria swarming effect under plasmonic optical fiber illumination
Source: J Biomed Opt. 2023 Jul 18;28(7):075003. doi: 10.1117/1.JBO.28.7.075003 (PMC10353699; doi:10.1117/1.JBO.28.7.075003)
Supplement: Supplementary file 1 [file JBO_028_075003_SD001.pdf]

Supplementary Materials

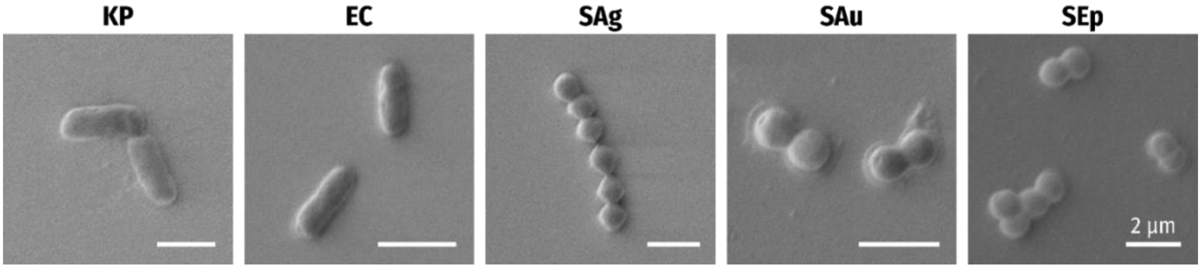

Fig. S1 SEM images of bacteria examined in this work. KP: *Klebsiella pneumoniae*; EC: *Escherichia coli*; SAg: *Streptococcus agalactiae*; SAu: *Staphylococcus aureus*; SEp: *Staphylococcus epidermidis*. Bacterial cells for imaging were deposited on silicon substrates and fixed using glutaraldehyde, followed by gradual dehydration using ethanol. Scale bars: 2 µm.

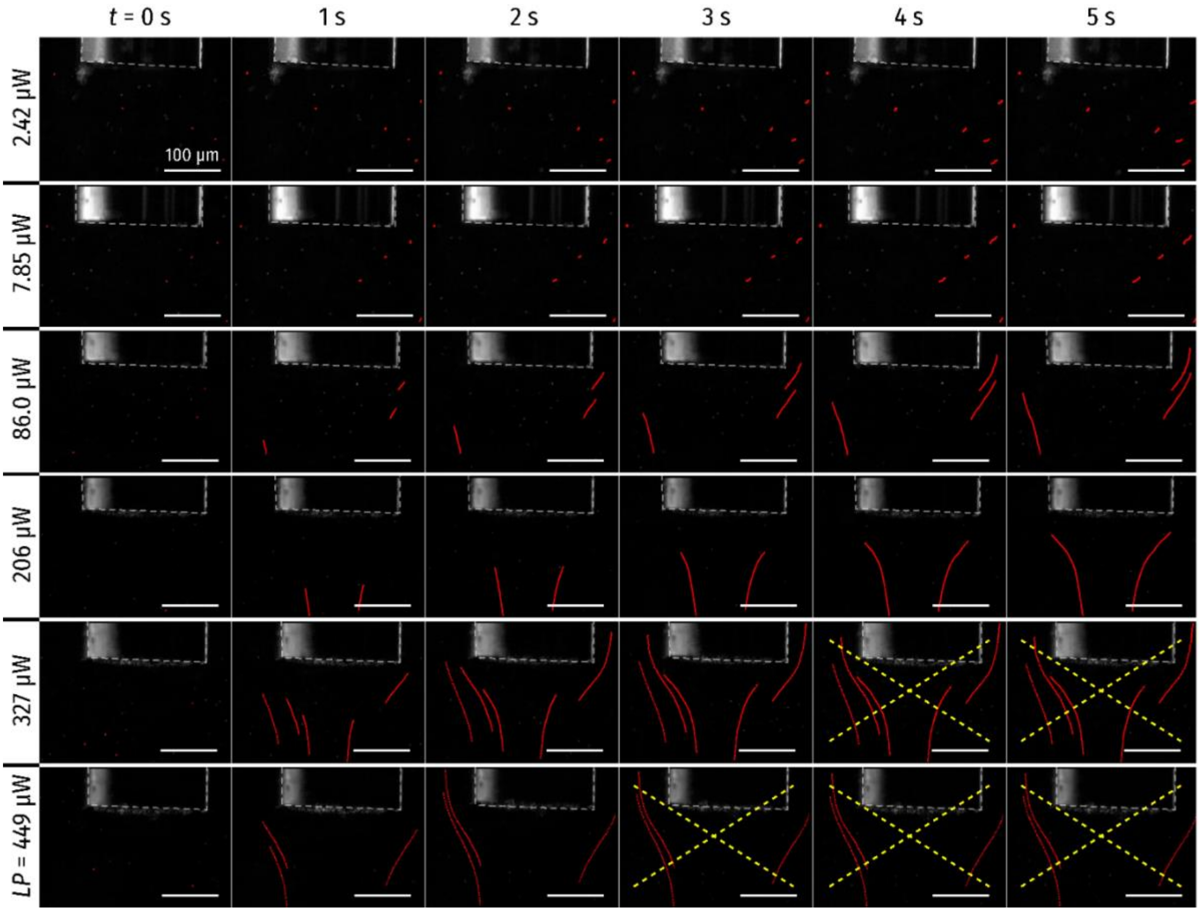

Fig. S2 Particle movement trajectory with various laser power (LP). PS diameter = 1.1 µm. Objective magnification = 1000x. Images including the trajectories of particles flowed out of the frame are discarded for the particle motion analysis (crossed images). Scale bars = 100 µm.

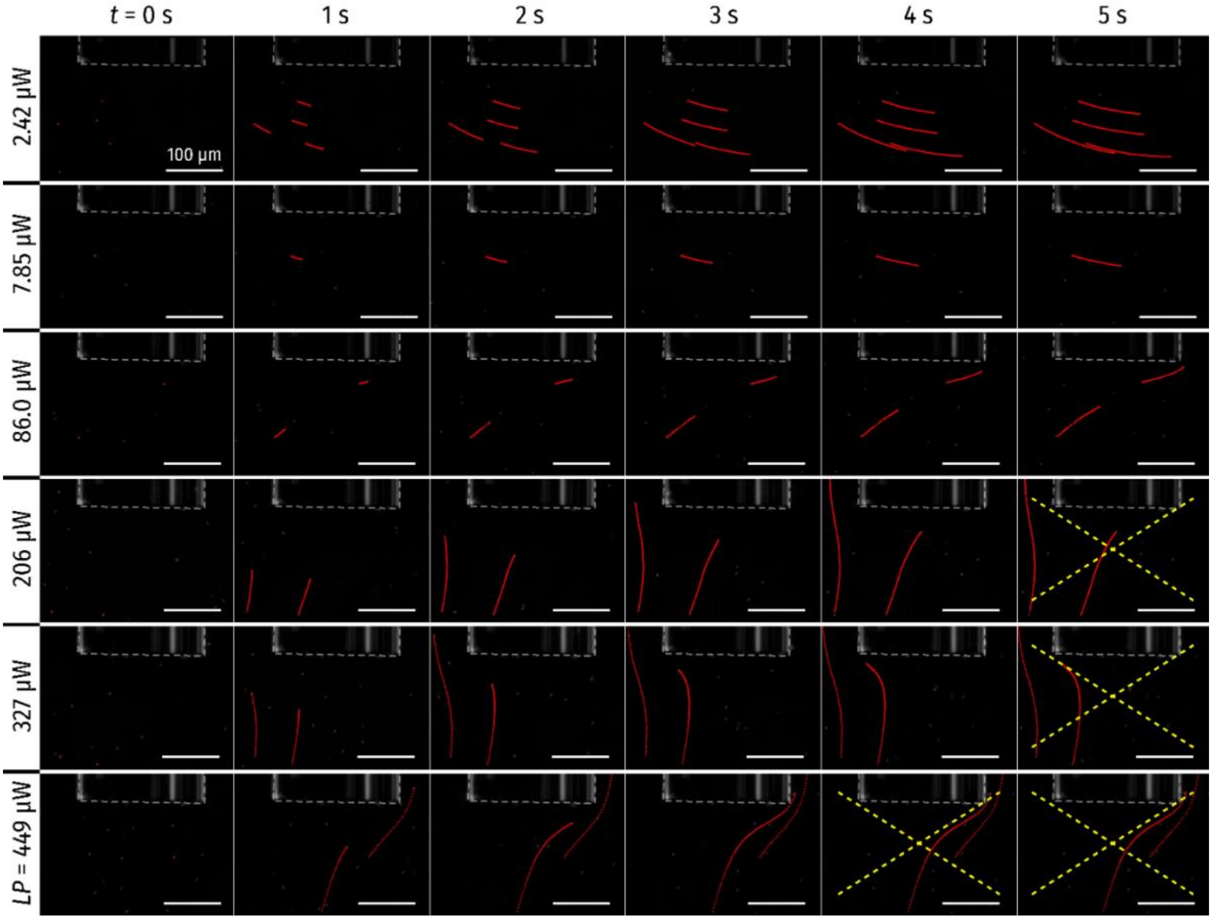

Fig. S3 Particle movement trajectory with various laser power (LP). PS diameter = 5 μm. Objective magnification = 1000x. Images including the trajectories of particles flowed out of the frame are discarded for the particle motion analysis (crossed images). Scale bars = 100 μm.

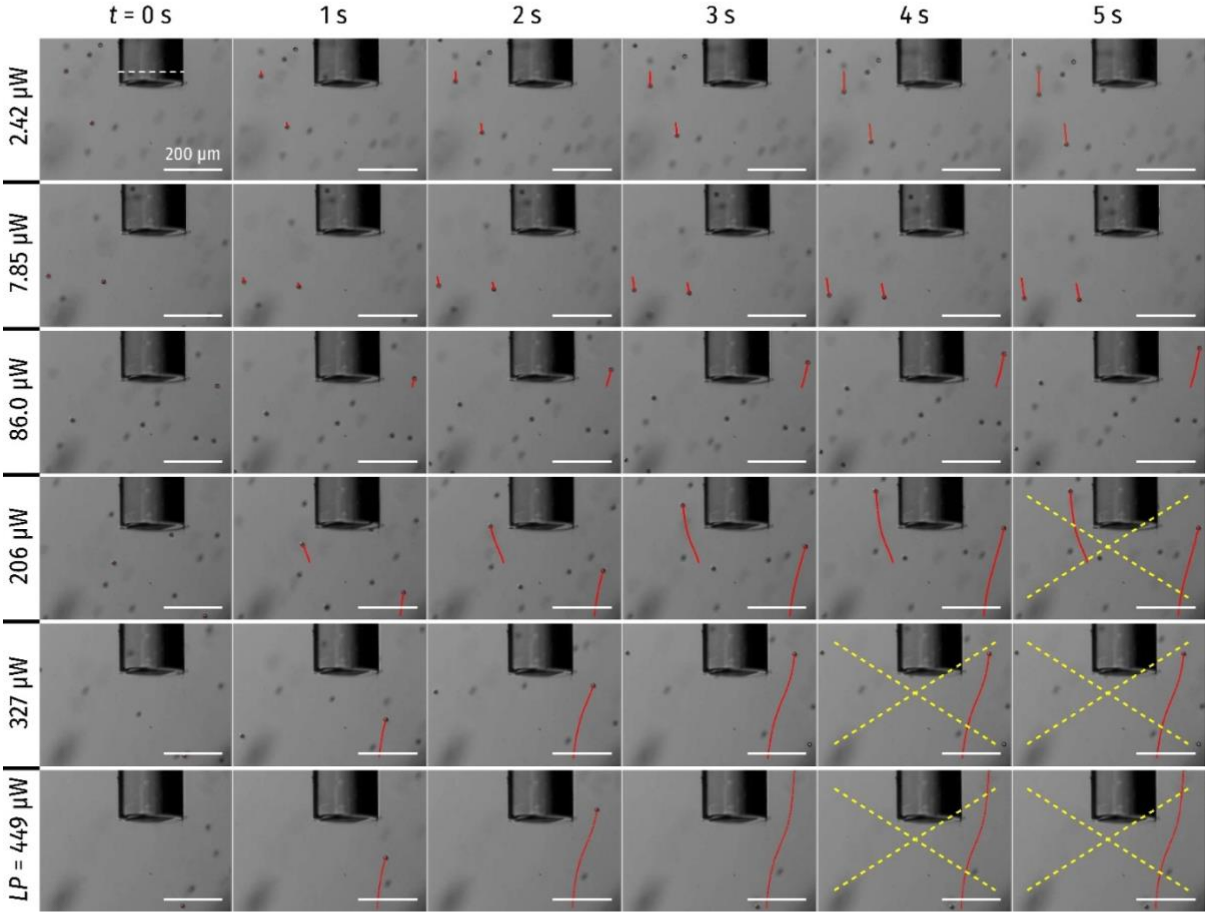

Fig. S4 Particle movement trajectory with various laser power (LP). PS diameter = 15 μm. Objective magnification = 500x. Images including the trajectories of particles flowed out of the frame are discarded for the particle motion analysis (crossed images). Scale bars = 200 μm.
